# Inguinal endometriosis: a case series and review of the literature

**DOI:** 10.1186/s13256-024-04400-x

**Published:** 2024-03-02

**Authors:** Ameneh Haghgoo, Ali Faegh, Seyyed Reza Saadat Mostafavi, Hamid Reza Zamani, Mehran Ghahremani

**Affiliations:** 1 School of Medicine, Nikan Hospital, Iran University of Medical Sciences, Aqdasiyeh, Army Boulevard, 22nd Bahman Street, Tehran, Iran; 2 School of Medicine, Alborz University of Medical Sciences, Karaj, Iran; 3 Department of Radiology, Hazrat-e-Rasoul Hospital, Iran University of Medical Sciences, Tehran, Iran; 4 Department of Radiology, Imam Hossein Medical and Educational Center, Shahid Beheshti University of Medical Sciences, Tehran, Iran; 5 Iranian Society of Pathology, Tehran, Iran

**Keywords:** Inguinal canal, Groin, Round ligament, Endometriosis, Inguinal hernia, Laparoscopy

## Abstract

**Background:**

Inguinal endometriosis is one of the most common forms of endometriosis. The present study introduces 8 cases of inguinal endometriosis and discusses probable theories of inguinal endometriosis by reviewing the literature.

**Case presentation:**

8 Iranian cases of inguinal endometriosis with a mean age of 36 years were presented. Catamenial groin pain and swelling were the most common complications. Also, patients usually had accompanying symptoms such as pelvic pain and dysmenorrhea. One-half of patients had a history of previous abdominal surgery. Ultrasound was diagnostic in 4 patients (50%), and magnetic resonance imaging was used in two patients (25%). Among 6 patients who underwent hormonal therapy, 4 experienced an endometriosis size increase. Inguinal endometriosis was right-sided in 87.5% of patients, and among 4 patients who underwent surgery, 75% had proximal site involvement of the round ligament.

**Conclusion:**

According to the rarity of inguinal endometriosis, it is more likely to be a misdiagnosis with other inguinal disorders such as inguinal hernia. Inguinal endometriosis should be considered in patients who undergo inguinal herniorrhaphy, with suspected findings such as thickening of the hernia sac wall, bloody fluid inside the sac, or thickening of the extraperitoneal round ligament during the surgery.

## Background

Endometriosis is a common gynecological disorder among women of reproductive age with about 10 to 15% prevalence [1, 2]. Ovaries are the most common site of endometriosis, although it can be identified in unconventional sites, such as the inguinal region, which accounts for 0.3–0.6% of all endometriosis [3–5]. 133 cases of inguinal endometriosis (IEM) have been reported until 2022 in the literature [6]. However, many inguinal IEM patients are not diagnosed because they undergo herniorrhaphy by general surgeons without any consideration for endometriosis [7]. IEM can co-exist with other groin disorders, such as inguinal hernia [8]. So, IEM is divided into three types: 1, IEM of inguinal hernia sac or hydrocele of canal of Nuck; 2, round ligament IEM; and 3, superficial IEM [9]. IEM’s diagnosis is challenging according to the wide range of differential diagnoses, such as inguinal hernia, inguinal lymphadenopathy, etc., so it can be misdiagnosed with other inguinal disorders commonly [10]. Female individuals with groin swelling or pain with menstruation-associated exacerbation (catamenial symptoms) should be assessed for IEM [6]. Although, a definitive diagnosis of IEM is usually made through histopathological examination [5]. Surgical resection is the standard treatment of IEM; nevertheless, the role of medical therapy is still controversial [10]. This study presents 8 cases of IEM who presented to the author’s office from 2017 to 2022. Also, it discusses probable theories of IEM by reviewing the literature.

## Case presentation

### Patient’s characteristics and symptoms

Eight Iranian cases of IEM, as well as one case of inguinal hernia with superficial implantations of the endometriosis surrounding the entrance of the hernia sac, were included^1^. The mean age was 36.3 years (standard deviation: 6.78, ranging between 30 and 51). Five patients were nulliparous, and the other 3 were multiparous. Visual analog scale (VAS) was used to assess the patient’s symptoms better. Patients with VAS ≥ 4 were considered to have the symptoms. Seven patients (87.5%) had complaints of inguinal swelling or pain, and six mentioned exacerbations in their menstrual period (catamenial symptoms). Also, seven patients (87.5%) had dysmenorrhea, 6 (75.0%) had dyspareunia, and 6 (75.0%) had pelvic pain.^1^

### History

Two patients had a history of primary infertility. Two had a history of inguinal herniorrhaphy, and four had a history of previous abdominal surgery. Also, six patients were diagnosed with pelvic endometriosis before presenting to the author’s office. The patient’s characteristics, symptoms, and history are summarized in Table 1.

**Table 1:** Patient’s characteristics and clinical information

N	Age	BMI	Parity	Groin mass or pain	Dysmenorrhea (VAS*)	Dyspareunia (VAS)	Pelvic pain (VAS)	Catamenial Symptom	CA-125*	History / previous ENM* Diagnosis	Medical treatment	Primary diagnosis	Imaging #Enzian classification	IEM* site	Surgical #Enzian classification
											Outcome	Follow-up (months)			
1	31	27.7	G1P1*	+	10	8	10	−	−	C-section*, appendectomy, and inguinal herniorrhaphy (1.2)/+	Progestin* GnRH agonist*	IEM*, adenomyosis	#Enzian_(u)_ O0/0, T2/0, B1/1, FA	Proximal / right RL*	P0, O0/0, T0/0, B0/0, F(RL)
											Size Pain	–			
2	30	20.7	N*	+	10	7	8	+	68.33	−/+	OCP*	IEM	#Enzian_(u)_ O0/0, T0/0, F(inguinal canal) #Enzian_(m)_ O0/0, T0/0, F(inguinal canal)	Distal/ right RL Closure to labia majora with Scarpa facia involvement	P1, O0/0, T0/0, B0/0, F(RL)
											Size Pain	–			
3	35	33.3	N	−	5	5	5	−	−	IVF*, PI*/oophorectomy, ENM* surgery/+	–	Severe PE	#Enzian_(u)_ O0/x, T1-/x-, B2/2, C3, FA	Proximal / right RL	P2, O1/0, T1/1, B3/2, F(RL)
												16			
4	41	17.5	N	+	4	−	0	+	18.50	−/+	Progestin GnRH agonist	IEM	#Enzian_(u)_ O0/0, T0/0, B1/1, F(inguinal canal)	Right RL	–
											Size Pain	24			
5	35	19.7	N	+	7	6	0	+	30.20	Pilonidal sinus cystectomy/+	Progestin	IEM, severe PE	#Enzian_(u)_ O0/0, T2/2, B2/2, C3, FI, F (inguinal canal)	Right Adhesion to right external iliac artery and vein	P0, O0/0, T0/0, B1/1, C3, F(RL)
											Size Pain	36			
6*	22	24.3	N	−	10	−	10	−	−	PI/+	Dydrogestrone*	Bilateral endometrioma	#Enzian_(u)_ O1/2, T0/0, B0/0	–	P3, O0/3, T1/0, B2/2, A3, FB, F(abdominal wall)
												40			
7	51	24.1	G3P3	+	3	10	10	+	−	NVD*, uterus polyp/−	GnRH agonist	Left IEM	#Enzian_(u)_ O0/0, T0/0, FA, F (left inguinal canal)	Left	–
											Size	18			
8	35	24.6	G3P3 Ab1EP1*	+	10	4	7	+	36.96	C-section, laparotomy (7), D&C* (10) (GA* = 13W*) /+	−	DE, IEM, endometrioma	#Enzian_(u)_ O1/1, T2/2, B2/1, C2, FI, F (inguinal canal)	Right	–
												–			
9	33	14.2	N	+	10	−	5	+	−	Inguinal herniorrhaphy (3)/−	Dienogest	IEM	#Enzian_(u)_ O0/0, T0/0, F (inguinal canal)	Right	–
											Size	36			

BMI: body mass index, VAS: Visual analog scale, CA-125: Cancer antigen-125, ENM: Endometriosis, G: gravida, P: para, N: Nulligravida HTN: hypertension, C-section: cesarean section, IEM: Inguinal Endometriosis, OCP: Oral Contraceptive pill, PI: Primary Infertility, IVF: *in-vitro* fertilization, NVD: normal vaginal delivery, Ab: abortion EP: ectopic pregnancy D&C: dilation and curettage, GA: gestational age, RL: Round ligament, #Enzian_(u)_: Ultrasound #Enzian score,, #Enzian_(m)_: MRI #Enzian score,, #Enzian_(s)_: Surgical #Enzian score

Progestin: (2 mg daily), GnRH agonist (10.8 mg, subcutaneous, every 12 weeks), Dydrogestrone (20 mg daily, from day 5 to day 25 of the menstrual cycle)

*Patient 6 has mild-size superficial endometriosis implantations surrounding the entrance of the inguinal hernia sac, which was identified accidentally during laparoscopic abdominal and pelvic exploration for endometriosis, but she had no IEM

### Physical examination and laboratory tests

Right and left inguinal mass was detected by physical examination in 6 patients (75.0%) and 1 patient (12.5%), respectively. Six patients had tenderness in the right groin and one on the left side. The Valsalva maneuver did not lead to an increase in the inguinal masse’s size. As shown in Table 1, the CA-125 value was measured in 4 patients. It was higher than the normal range in only 2 patients.

### Imaging assessment

**Fig. 1:**
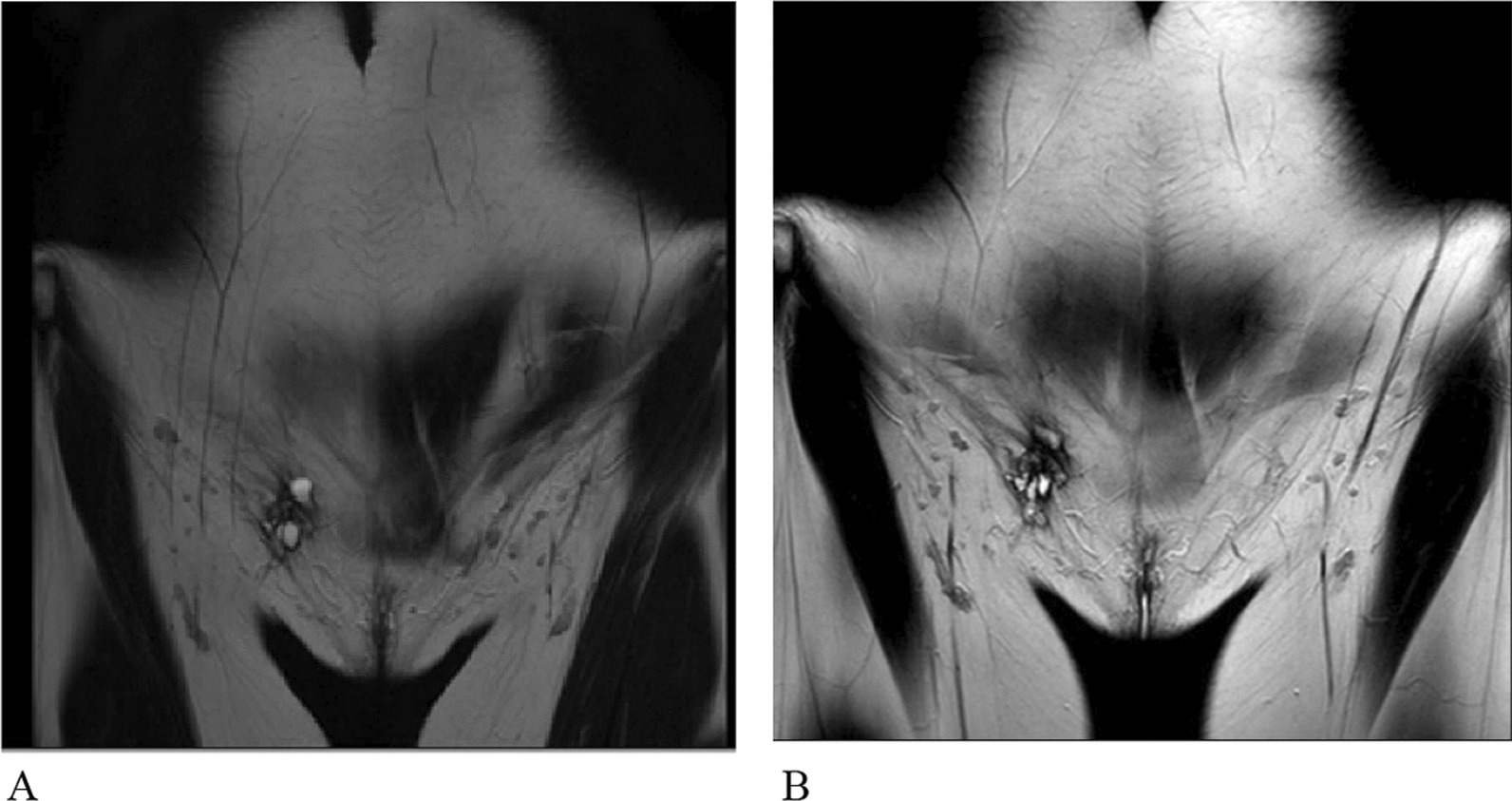
Comparison of inguinal endometriosis size in magnetic resonance imaging. **A** first magnetic resonance imaging (35 × 9 mm); **B** second magnetic resonance imaging 9 months later (61 × 60 mm)

Inguinal ultrasound revealed IEM in 6 patients. Trans-abdominal and transvaginal ultrasounds were performed on two patients because they have symptoms associated with pelvic endometriosis. Also, two patients (25.0%) were misdiagnosed previously by ultrasound, one with inguinal hernia and the other with inguinal lymphadenopathy. IEM size increase was assessed for 2 patients. In one patient, IEM size increased about 17 × 1 mm during 4 years with an average growth of (4 × 0.25 mm) per year despite medical treatment with progestin and GnRH agonists. In another patient, the IEM size was decreased, but notably, she underwent medical treatment with progestin during those 2 years. Also, two patients underwent magnetic resonance imaging (MRI). It revealed IEM in both patients. As shown in Fig. 1, MRI in patient 2 revealed 29 × 51 mm growths during 9 months despite taking an oral contraceptive. We used imaging #Enzian classification to describe imaging assessment results better. Imaging’s findings are summarized in Table 1.

### Treatment and surgical methods

As shown in Table 1, medical treatment by progestin, GnRH agonist, and Dydrogesterone was performed on 6 Patients, leading to IEM size decrease in 2 of them; however, IEM size increased in 4 patients, and their pain got worse.

Laparoscopic surgical intervention was performed on 5 patients. Four underwent IEM resection surgery. Under general anesthesia, they secured a Trendelenburg position. A central umbilical trocar (12 mm), 2 lateral trocars (5 mm), and a suprapubic trocar (5 mm) were inserted. Intraoperative CO_2_ pressure was established at 13 mm Hg. First, endometriosis abdominal and pelvic exploration was performed. Then, inguinal canal exploration was performed. Inguinal mass was resected with a safe margin in these patients. In addition, in patient 5, IEM was adherent to the femoral, external iliac, and inferior epigastric vessels. So, surgery was performed by a multidisciplinary team, including an expert vascular surgeon and a gynecologist. IEM was resected with a safe margin without any vascular injury. Then, a skin incision was conducted in the groin. In patients with involvements, IEM mass was resected from the external oblique, transversus abdominis, internal oblique muscles, and Scarpa facia. IEM lesion and resected part of the round ligament were pulled out via a skin incision. The abdominal wall defect was repaired by Prolene 3-0 suture in all patients. Also, to avoid an incisional hernia, a PROLENE Mesh was placed on the inguinal canal and fixed with Prolene 3-0 suture for patient 5. Also, a Hemovac drain was established to prevent post-surgical collection in this patient. After abdominal wall repair, the peritoneal defect was repaired via laparoscopy to avoid gas leakage, subcutaneous emphysema formation, and intestinal hernia. In cases with co-existing inguinal hernia, Inguinal hernia sacs and IEM were resected, and the abdominal wall defects were repaired. Then, abdominal-pelvic laparoscopic exploration was performed, and endometriosis lesions were removed in patients with other endometriotic lesions.

**Fig. 2:**
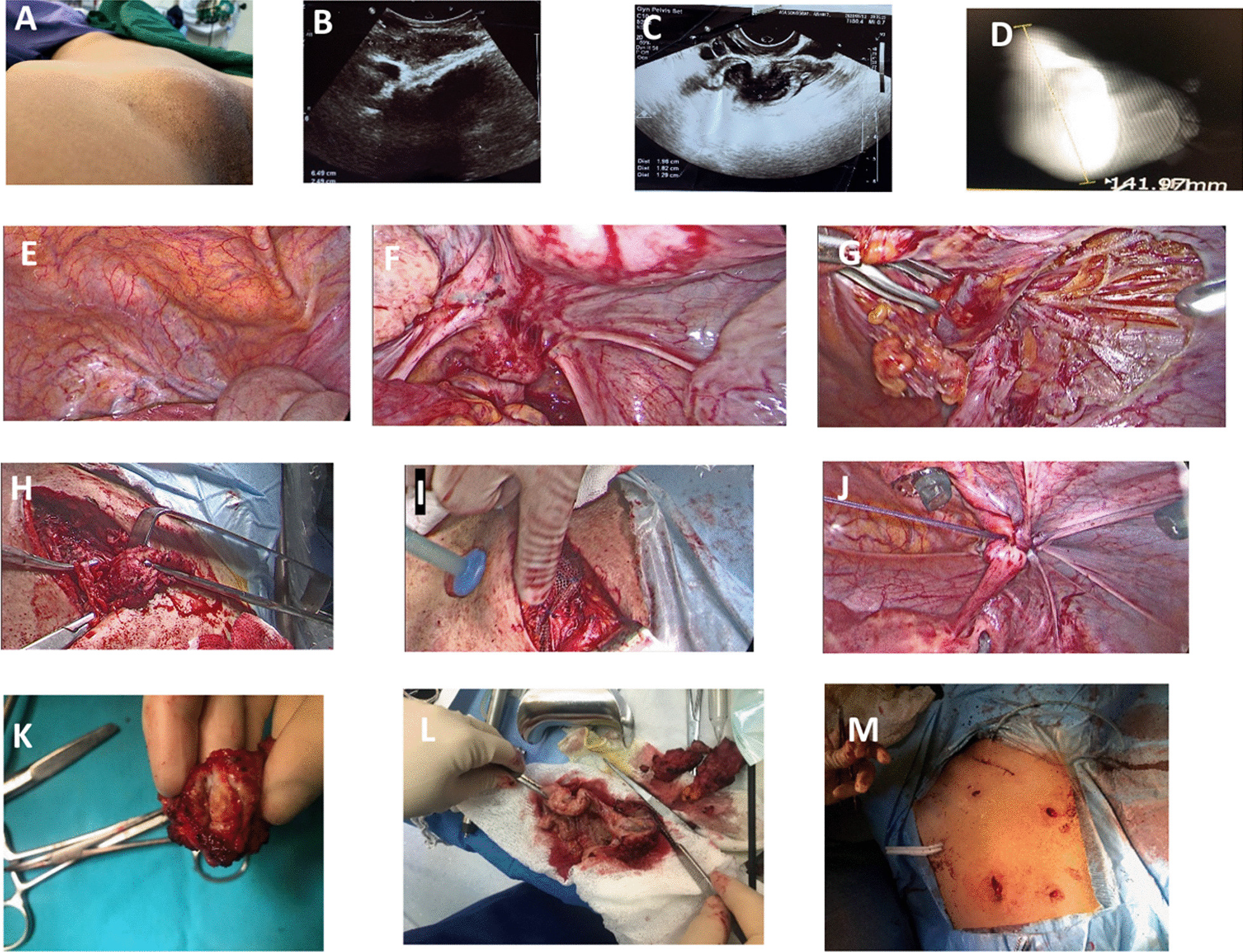
Pre-operative imaging assessment and intraoperative findings of case 5. **A** groin palpable mass; **B** inguinal endometriosis in ultrasound; **C** rectum endometriosis in transvaginal ultrasound; **D** inguinal endometriosis in magnetic resonance imaging; **E** inguinal bulging was seen during abdominal exploration; **F** uterosacral ligament endometriosis; **G** round ligament, external iliac artery, and vein after making incision; **H** pulling out inguinal mass via skin incision after resection with safe margin; **I** placing PROLENE Mesh on the external orifice of the inguinal canal; **J** peritoneal defect repairment; **K** inguinal endometriosis; **L** rectum endometriosis and inguinal mass; **M** Hemovac drain on inguinal site and corrugated drain on the anastomosis

**Fig. 3:**
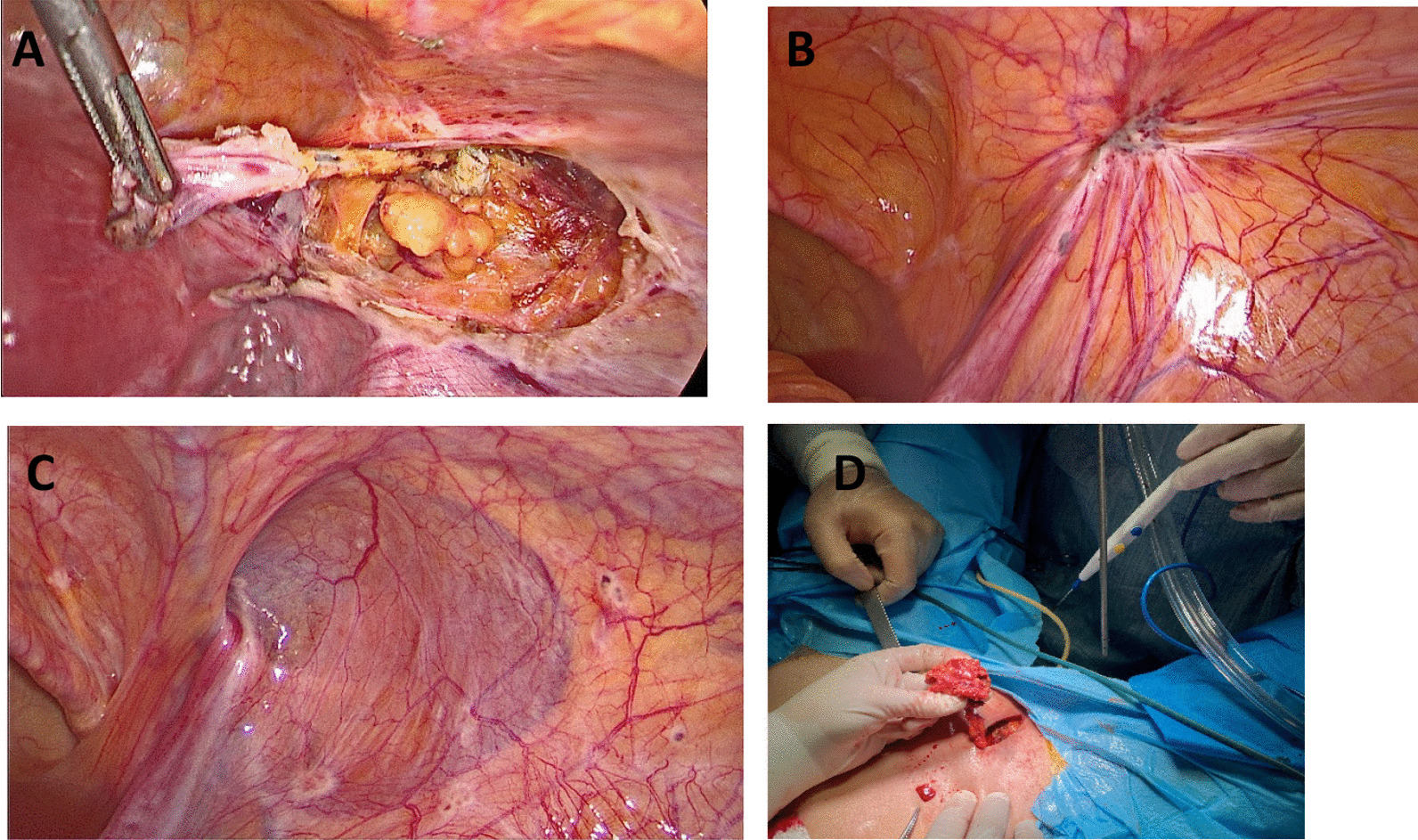
Abdominal view of inguinal endometriosis and pulling out inguinal mass via skin incision. **A** inguinal canal and round ligament involvement by endometriosis (patient 1); **B** endometriosis implantations on the internal orifice of the inguinal canal (patient 3); **C** superficial endometriosis surrounding the entrance of inguinal hernia sac (patient 6); **D** inguinal endometriosis and round ligament remove via skin incision (patient 2)

Patient 6 underwent pelvic endometriosis laparoscopic surgery due to pelvic endometriosis diagnosis. Nevertheless, mild-size endometrioid implantations were identified accidentally surrounding the entrance of the inguinal hernia sac during laparoscopy (Figs. 2 and 3). As mentioned, she had no IEM, so she was excluded from statistical analysis.

### Surgical findings

Among patients who underwent surgical interventions for IEM, three patients (75%) had proximal part of the inguinal canal and round ligament involvement by IEM. However, in one patient (25%), the proximal part was not involved, and IEM involved the distal portion of the round ligament near the labia majora and pubic symphysis. Also, two had co-existing inguinal hernia. The surgical #Enzian classification was used to better describe the patients’ surgery findings. Surgical interventions and findings are summarized in Table 1.

### Histopathological examination and follow-up

**Fig. 4:**
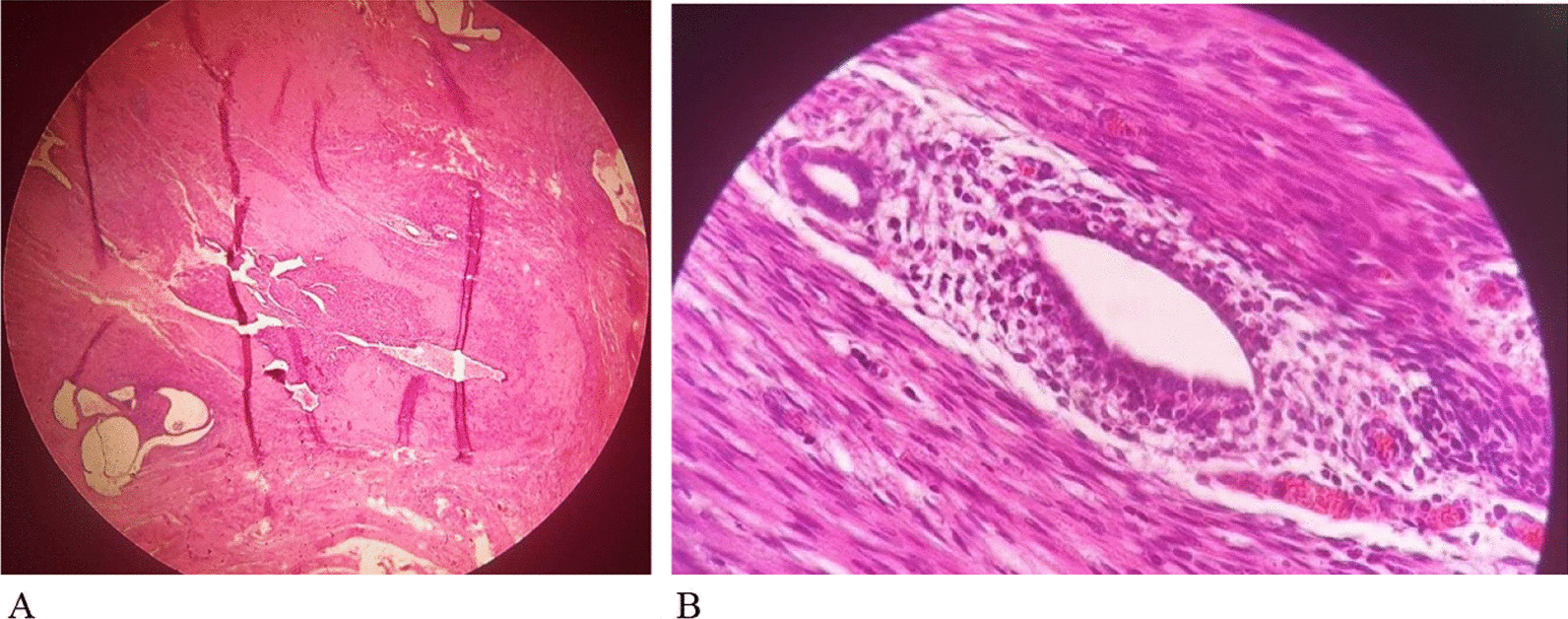
Histopathological appearance of inguinal endometriosis. **A** glandular and stromal endometrial tissue surrounded by fibromuscular tissue × 40 LPF; **B** glandular and stromal endometrial tissue surrounded by fibromuscular tissue × 400 HPF

Histopathology examination revealed IEM in all four patients who had inguinal lesions. Also, it demonstrated abdominal wall endometriosis for patient 6 (Fig. 4).

As shown in Table 1, during a long-time follow-up (> one year) for five patients who underwent surgery, patients had no complaints and suspected symptoms of IEM recurrence. The results of the statistical analysis are summarized in Table 2.

**Table 2:** Clinical data of this study

Parameter/ Total cases	Number of cases and percentage *n* (%)				
Parity/8	Nulliparous 5 (62.5)	Multiparous 3 (37.5)			
Symptoms/8	Dysmenorrhea 7 (87.5)	Pelvic pain 6 (75.0)	Dyspareunia 6 (75.0)	Groin mass or pain 7 (87.5)	Catamenial groin pain 6 (75.0)
CA-125*/4	Normal range 2 (50.0)	High 2 (50.0)			
Gynecologic history/8	NVD* 1 (12.5)	C-section* 2 (25.0)		Endometriosis 6 (75.0)	
Previous abdominal surgery/8	Yes 4 (50.0)	No 4 (50.0)		Herniorrhaphy 2 (25.0)	
IEM* diagnosis/8	Ultrasound 4 (50.0)	Ultrasound and MRI* 2 (25.0)		Accidentally, during the surgery 1 (12.5)	Misdiagnosis by ultrasound 2 (25.0)
Treatment/8	Medical Treatment 6 (75.0)	Medical Treatment Outcome (total 6) Increased size: 4 (66.6) Decreased size: 2 (33.3)		Surgical Treatment 4 (50.0)	
IEM laterality/8	Right 7 (87.5)	Left 1 (12.5)			
IEM site in patients who underwent surgery/4	Proximal 3 (75.0)	Distal 1 (25.0)		Co-existing IEM and inguinal hernia 2 (50.0)	
Other sites endometriosis/8	Endometrioma 2 (25.0)	Deep endometriosis 4 (50.0)			

*CA-125: Cancer antigen-125; NVD: normal vaginal delivery; C-section: Cesarean section, IEM: inguinal endometriosis

## Discussion and conclusion

### Clinical manifestations and differential diagnoses

This study discussed 8 cases of IEM with a mean age of 36. Five were nulliparous, so it seems there is no relationship between IEM and parity history. Catamenial groin pain or swelling was the most frequent symptom. Also, patients usually had accompanying symptoms such as pelvic pain and dysmenorrhea. One-half of patients had a history of previous abdominal surgery. Ultrasound was diagnostic in 4 patients (50%), and MRI was used in two patients (25%). Among 6 patients who underwent hormonal therapy, 4 (66.6%) experienced IEM size increase. IEM was right-sided in 87.5% of patients, and among 4 patients who underwent surgery, 75% had proximal site inguinal involvement.

About 133 cases of IEM were reported in the literature until 2022. The mean age is about 36 years old, and the largest number of the reported instances belongs to Japan [6]. Right-side IEM is more frequent than left-side; therefore, right inguinal swelling with pain exacerbation in the menstrual cycle is the typical clinical manifestation. Nevertheless, cases of bilateral or left-side IEM and cases without catamenial symptoms were reported [6]. Dysmenorrhea, dyspareunia, and pelvic pain are accompanying symptoms. However, in patients without these symptoms, IEM is not a diagnosis of exclusion [11]. Also, about 30% of IEM cases have a previous operation [6].

There are various causes for an inguinal mass, such as inguinal hernia, hydrocele of the canal of Nuck, inguinal lymphadenopathy, hemangioma, and malignancies [12–14]. Also, endometrial-like tissue can be identified in the sac of the inguinal hernia or the hydrocele of the canal of Nuck [13, 15]. So, about 20% of patients with IEM have Co-existing inguinal hernia or canal of Nuck hydrocele [6]. Several cases of IEM are misdiagnosed as an inguinal hernia due to the greater prevalence of inguinal hernia, less consideration of general surgeons for endometriosis, and insufficient post-operative histopathological assessments [10]. According to the wide range of differential diagnoses of inguinal swelling, diagnosis of the IEM is challenging and requires careful investigations. MRI is the most sensitive and specific imaging modality for IEM diagnosis. MRI identifies iron particles in the hemosiderin, in contrast with computed tomography. Therefore, MRI has more sensitivity and specificity than computed tomography. MRI shows IEMs as hyperechoic lesions on T1 images and hypoechoic on T2 images [11, 16–18]. However, ultrasound is valuable and diagnostic in most patients [6, 13, 19]. Abdominal ultrasound has been used to diagnose IEM in about 74% of patients [6]. Ultrasound is an appropriate modality for detecting cystic peritoneal implants in contrast to small peritoneal implantations. Still, the sensitivity and specificity of the ultrasound for the diagnosis of round ligament endometriosis have not been evaluated due to the limited number of studies [20].

A high CA-125 level is not identified in all cases of IEM, even though there are several cases of IEM with increased CA-125 [6]. Two patients (25%) among our study population had CA-125 levels higher than the normal range. In one of them, the CA-125 level was near the upper normal limit (36.96 U/Ml), and only one had a CA-125 high level (12.5%). In highly suspected cases of IEM, fine-needle aspiration cytology can confirm the IEM diagnosis before the operation, but it is not performed commonly [17].

### Pathophysiology

Some theories describe the pathophysiology of endometriosis, including retrograde menstruation (Sampson theory), lymphatic or hematogenous benign metastasis, and Mullerianosis theory [21–23]. Dissemination of endometrial cells along the round ligament from the abdominal cavity is the more acceptable theory in cases with co-existing pelvic endometriosis. In these patients, endometriosis usually can be seen in the proximal part of the round ligament and inguinal canal [10]. Although, in isolated IEM without pelvic endometriosis, IEM nodules are usually seen on the distal part of the round ligament and not on the proximal portion. We hypothesize that Mullerianosis seems to be the most favorable theory in these patients. The round ligament involvement pattern can help to describe the appropriate theory in each patient better. Also, we hypothesize that the isolated distal part of the round ligament involvement by endometriosis is against retrograde menstruation theory and advocates for the Mularianosis theory in patients without co-existing pelvic endometriosis.

Peritoneal fluid circulates clockwise because of gravity and diaphragm respiratory movements [24]. Although, endometrial free cells in the peritoneal fluid spread in the abdominal cavity. The sigmoid colon prevents the entrance of the peritoneal fluid to the left inguinal canal [10]. The right-side canal does not have this support, so the round ligament plays a role as a transmitter of endometrial cells to the groin. It can be an appropriate explanation for IEM cases with co-existing pelvic endometriosis [10]. According to these two facts, the right-side dominancy of the IEM can be described [25].

### Treatment

Surgical excision of the inguinal mass with inguinal canal exploration is the most common treatment [6]. The operation can be achieved by both open and laparoscopic approaches [26]. Due to the proximity of IEM lesions, external iliac and femoral vessels, complete resection of the inguinal mass with a safe margin without vessel wall damage is the most critical issue that should be considered during surgical excision. In case of co-existing inguinal hernia or canal of Nuck hydrocele, excision of the hernia sac and abdominal wall repairment by Mesh is required [10, 26]. Laparoscopic pelvic and abdominal cavity exploration is probably needed in case of co-existing pelvic or abdominal endometriosis. Patients who undergo inguinal herniorrhaphy with suspected findings (such as thickening of the hernia sac wall, bloody fluid inside the sac, or thickening of the extraperitoneal round ligament) during the surgery by general surgeons should refer to gynecologists for more assessments about other sites of endometriosis [10].

The sufficiency of hormonal therapy for IEM is not strongly acceptable and requires more investigation due to the limited number of patients undergoing medical treatment [6]. Post-operation hormonal therapy is often performed to prevent endometriosis recurrence [27]. Nevertheless, despite the slight recurrence rate of IEM, the benefits of hormonal treatment should be investigated [6]. However, for patients with IEM, enough time should be considered to choose the best surgical team and plan due to the low velocity of IEM growth.

Definitive diagnosis of IEM is based on post-operative histopathological examination [5]. Post-operative histopathological examination is crucial due to variations of histopathological findings, such as uterus-like tissue and malignant clear-cell carcinoma [6, 28].

In conclusion, IEM is a rare condition requiring precise diagnosis assessments. In case of an inguinal mass with aggravated swelling or pain in the menstrual period, IEM should always be considered. MRI is the most sensitive modality of IEM diagnosis. Also, ultrasound has been used for diagnosis. However, because ultrasound is an operator-dependent modality, suspected patients for IEM should be referred to an expert sonologist with enough experience in endometriosis diagnosis. Due to a wide range of differential diagnoses of groin masses, IEM can be misdiagnosed commonly. A more precise post-operative histopathological examination is required about inguinal masses. In cases that undergo inguinal herniorrhaphy and diagnosis with IEM during or after the operation, a gynecological consultant is necessary to investigate the other sites of endometriosis.

## Data Availability

Further information about this study is available by contacting the corresponding author.

## Abbreviations

IEM: Inguinal endometriosis
VAS: Visual analog scale
MRI: Magnetic resonance imaging

## Footnotes

1 Patient 6 has mild-size superficial endometriosis implantations surrounding the entrance of the inguinal hernia sac, which was identified accidentally during laparoscopic abdominal and pelvic exploration for endometriosis, but she had no IEM, so she was excluded, and then statistical analysis was performed on 8 patients with IEM.

## References

[1] de Graaff AA, D’Hooghe TM, Dunselman GAJ, Dirksen CD, Hummelshoj L, Simoens S (2013). The significant effect of endometriosis on physical, mental and social wellbeing: results from an international cross-sectional survey. Hum Reprod.

[2] Mehedintu C, Plotogea MN, Ionescu S, Antonovici M (2014). Endometriosis still a challenge. J Med Life.

[3] Lee HJ, Park YM, Jee BC, Kim YB, Suh CS (2015). Various anatomic locations of surgically proven endometriosis: a single-center experience. Obstet Gynecol Sci.

[4] Blanco RG, Parithivel VS, Shah AK, Gumbs MA, Schein M, Gerst PH (2003). Abdominal wall endometriomas. Am J Surg.

[5] Jena SK, Begum J, Kumari S, Kar C (2020). The groin endometriosis: a great mimicker of common groin conditions. J Gynecol Surg.

[6] Dalkalitsis A, Salta S, Tsakiridis I, Dagklis T, Kalogiannidis I, Mamopoulos A (2022). Inguinal endometriosis: a systematic review. Taiwan J Obstet Gynecol.

[7] Zihni İ, Karaköse O, Özçelik KÇ, Pülat H, Eroloğlu HE, Bozkurt KK (2020). Endometriosis within the inguinal hernia sac. Turk J Surg.

[8] Prodromidou A, Pandraklakis A, Rodolakis A, Thomakos N (2020). Endometriosis of the canal of nuck: a systematic review of the literature. Diagnostics.

[9] Niitsu H, Tsumura H, Kanehiro T, Yamaoka H, Taogoshi H, Murao N (2019). Clinical characteristics and surgical treatment for inguinal endometriosis in young women of reproductive age. Dig Surg.

[10] Li S-H, Sun H-Z, Li W-H, Wang S-Z (2021). Inguinal endometriosis: ten case reports and review of literature. World J Clin Cases.

[11] Hagiwara Y, Hatori M, Moriya T, Terada Y, Yaegashi N, Ehara S (2007). Inguinal endometriosis attaching to the round ligament. Australas Radiol.

[12] Yang DM, Kim HC, Ryu JK, Lim JW, Kim GY (2010). Sonographic findings of inguinal endometriosis. J Ultrasound Med.

[13] Cervini P, Mahoney J, Wu L (2005). Endometriosis in the canal of nuck: atypical manifestations in an unusual location. Am J Roentgenol.

[14] Fujikawa H, Uehara Y. Inguinal endometriosis: unusual cause of groin pain. Balkan Med J. 2020; 10.4274/balkanmedj.galenos.2020.2020.2.105 PMC7424186 32212577

[15] Albutt K, Glass C, Odom S, Gupta A (2014). Endometriosis within a left-sided inguinal hernia sac. J Surg Case Rep.

[16] Licheri S, Pisano G, Erdas E, Ledda S, Casu B, Cherchi MV (2005). Endometriosis of the round ligament: description of a clinical case and review of the literature. Hernia.

[17] AlSinan FM, Alsakran AS, Foula MS, al Omoush TM, Al-Bisher H. Inguinal endometriosis in a nulliparous woman mimicking an inguinal hernia: a case report with literature review. Am J Case Rep. 2021;22. 10.12659/AJCR.934564 PMC8693242 34916480

[18] Gaeta M, Minutoli F, Mileto A, Racchiusa S, Donato R, Bottari A (2010). Nuck canal endometriosis: MR imaging findings and clinical features. Abdom Imaging.

[19] Kamkarfar P, Shahriyaripoor R, Rokhgireh S, Mostafavi SRS, Chaichian S, Mehdizadeh KA (2022). Comparison of diagnostic values of transvaginal sonography with laparoscopic and histological results in the evaluation of uterosacral ligaments’ involvement in endometriosis patients. Caspian J Intern Med.

[20] Bazot M, Daraï E (2017). Diagnosis of deep endometriosis: clinical examination, ultrasonography, magnetic resonance imaging, and other techniques. Fertil Steril.

[21] Sampson JA (1927). Peritoneal endometriosis due to the menstrual dissemination of endometrial tissue into the peritoneal cavity. Am J Obstet Gynecol.

[22] Pergialiotis V, Lagkadas A, Polychronis O, Natsis S, Karakalpakis D, Giannakopoulos K (2011). Endometriosis-associated ovarian cancer. Presentation of a case report and review of the literature. Eur J Gynaecol Oncol.

[23] Batt RE, Yeh J (2013). Müllerianosis: four developmental (embryonic) mullerian diseases. Reprod Sci.

[24] Sun Z-J, Zhu L, Lang J-H (2010). A rare extrapelvic endometriosis: inguinal endometriosis. J Reprod Med.

[25] Burcharth J (2014). The epidemiology and risk factors for recurrence after inguinal hernia surgery. Dan Med J.

[26] Hwang B, Bultitude J, Diab J, Bean A. Cyst and endometriosis of the canal of Nuck: rare differentials for a female groin mass. J Surg Case Rep. 2022;2022. 10.1093/jscr/rjab626 PMC8784177 35079343

[27] Lee SE, Jo DH, Moon SH, Chong HI, Shin SI, Kim HG (2008). A case of inguinal endometriosis in the absence of previous gynecologic surgery. Korean J Obstet Gynecol..

[28] Seki A, Maeshima A, Nakagawa H, Shiraishi J, Murata Y, Arai H (2011). A subserosal uterus-like mass presenting after a sliding hernia of the ovary and endometriosis: a rare entity with a discussion of the histogenesis. Fertil Steril.

